# Spatial and chronological patterns of the lithics of hearth 1 at the Gravettian site Krems-Wachtberg

**DOI:** 10.1016/j.quaint.2011.10.031

**Published:** 2014-11-17

**Authors:** Roswitha Thomas, Johanna Ziehaus

**Affiliations:** Austrian Academy of Sciences, Prehistoric Commission, Fleischmarkt 22, 1010 Vienna, Austria

## Abstract

A spatial and micro-stratigraphic interpretation of the Gravettian open-air site Krems-Wachtberg in Lower Austria uses analysis of the lithic artefacts of an area of the excavations. The results are based on conclusive aspects of the artefact morphology and raw material attribution. Investigations focus on the acquisition of the spatial structure of living floor AH (archaeological horizon) 4.4 with a well-preserved hearth. Different utilization phases of hearth 1 with their relationships to the surrounding areas are discussed and a comparison of the in situ AH 4.4 and the post-occupational deposition of AH 4.11 presented. The examination of the artefact typology supports an attribution to the Early Gravettian of Central Europe (Pavlovian, 30–24 ka BP).

## 1 Introduction

The Gravettian open-air site Krems-Wachtberg, situated on the northern bank of the Danube in north-east Austria, has been excavated since 2005 (see Simon et al., 2014). The site became known for its two infant burials, one double grave with two newborns and a single grave with a three-month old individual, which are embedded in a very well-preserved living floor with a hearth. A range of different publications has been published on recent results of the Krems-Wachtberg excavations (Einwögerer et al., 2006, Einwögerer et al., 2008, Einwögerer et al., 2009, Händel et al., 2009a, Händel et al., 2009b, Hambach, 2014).

The archaeological investigations focussed on the anthropogenic stratigraphic complex AH (archaeological horizon) 4. The main units are represented by a translocated layer AH 4.11 which is situated directly above a living floor AH 4.4.

Due to observations during the excavations, it can be assumed that the upper parts of AH 4.4 had been eroded and only its base remained in situ. AH 4.11 consists of the eroded part of AH 4.4 and material from an unknown source uphill (Händel et al., 2009a, 189–190; Händel et al., 2009b, 47). This will be discussed below, for the part of the excavated area including the hearth, considering selected aspects of morphology, raw material and distribution of lithic artefacts investigated to date.

An important feature within AH 4.4 is the multi-phased hearth 1. It has been divided in three main phases, with each consisting of several sediment units (Simon et al., 2014). The upper part of the underlying loess is burnt due to heat exposure. Phase I shows a fragmentarily preserved layer of stone plates. Remains of a similar layer of slab fragments were found at the base of phase II, but less preserved than in phase I. The hearth was intentionally enlarged to the southeast in phase II. Some areas showed patches of burnt loess at the base of the final phase III. The last phase contains a lower amount of charcoals and therefore a higher percentage of burnt bones, which suggests a change in firing material.

Besides chert artefacts, other remains were found in the hearth. Small fragments of “ceramics” (formed and fired sediment) occur especially in phase I, including a figurine (Händel et al., 2009a; Fig. 7).

**Fig. 1:**
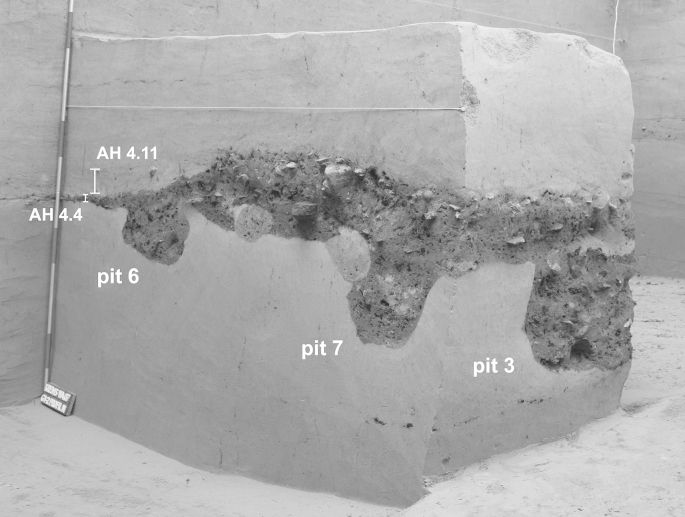
Krems-Wachtberg: Hearth 1 with adjoining pits (photo: Prehistoric Commission, Austrian Academy of Sciences).

**Fig. 2:**
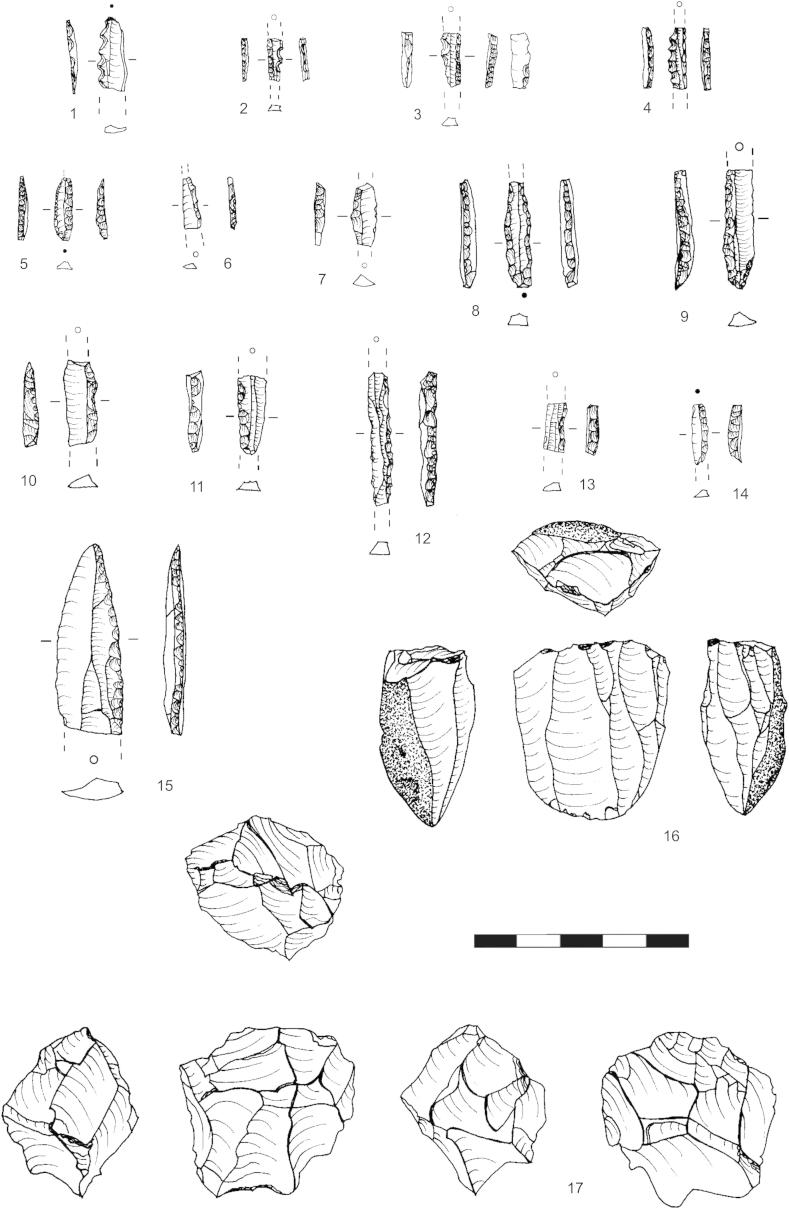
Krems-Wachtberg: 1–4 microdenticulates, 5 microlith, 6–9 microgravettes, 10–14 backed bladelets, 15 backed point, 16 unidirectional blade core, 17 rotated flake core (graph: Prehistoric Commission, Austrian Academy of Sciences).

**Fig. 3:**
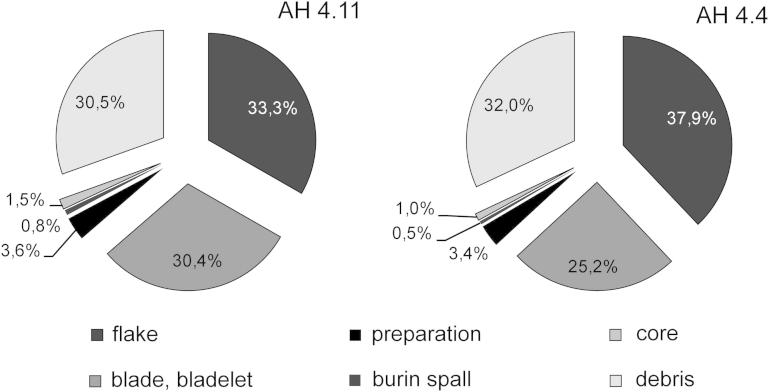
Krems-Wachtberg: Lithic debitage in layers AH 4.4 and AH 4.11 (graph: Prehistoric Commission, Austrian Academy of Sciences).

**Fig. 4:**
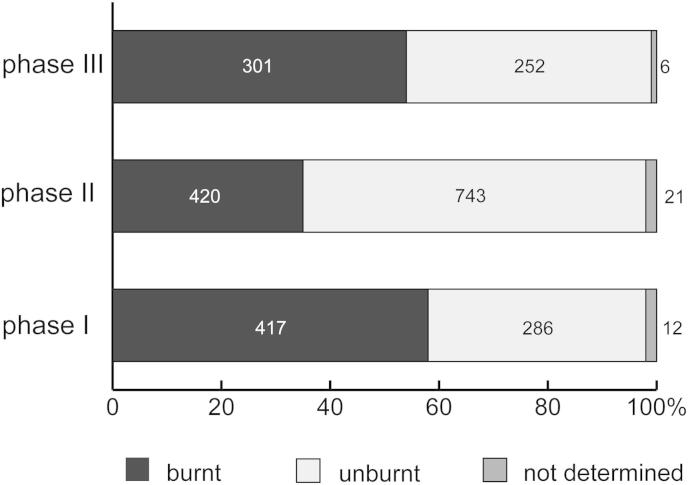
Krems-Wachtberg: Lithic debitage with fire impact per phase in hearth 1 (graph: Prehistoric Commission, Austrian Academy of Sciences).

**Fig. 5:**
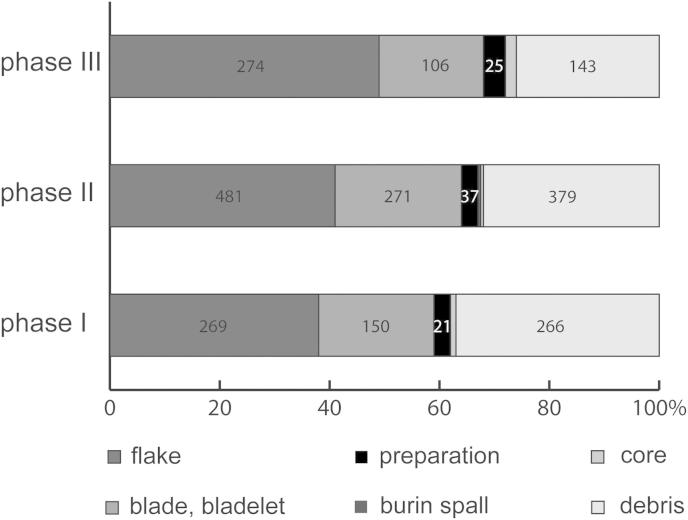
Krems-Wachtberg: Lithic debitage per phase in hearth 1 (graph: Prehistoric Commission, Austrian Academy of Sciences).

**Fig. 6:**
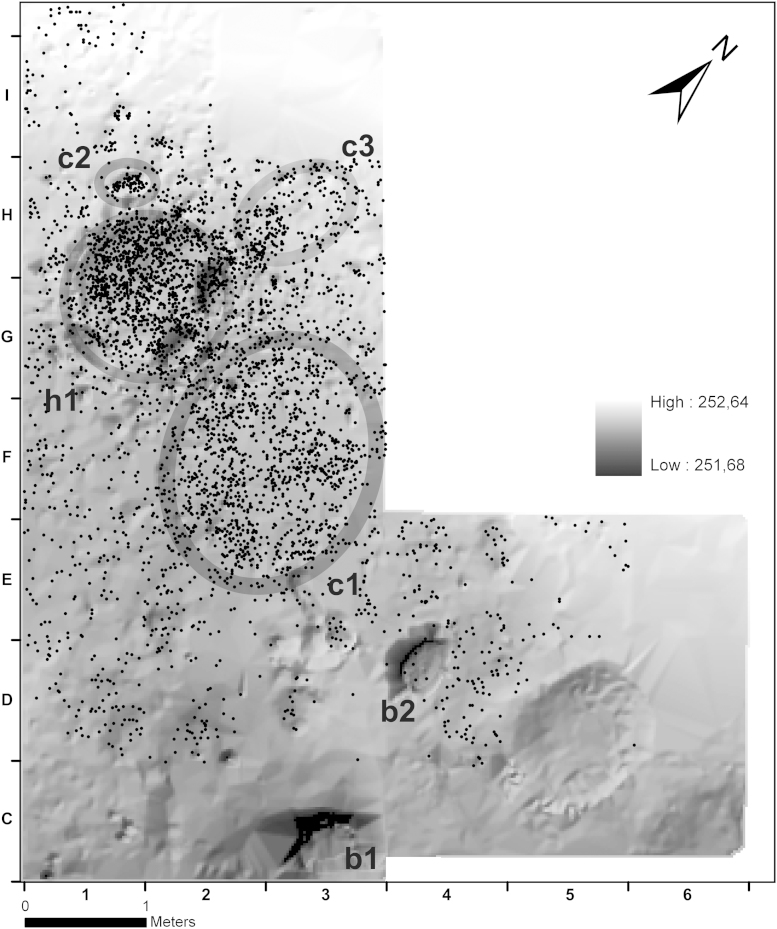
Krems-Wachtberg: Surface model with spatial distribution of lithic artefacts in layer AH 4.4: h1 – hearth 1 complex, b1–2 – burial 1–2, c1–3 – concentration 1–3 (graph: Prehistoric Commission, Austrian Academy of Sciences).

**Fig. 7:**
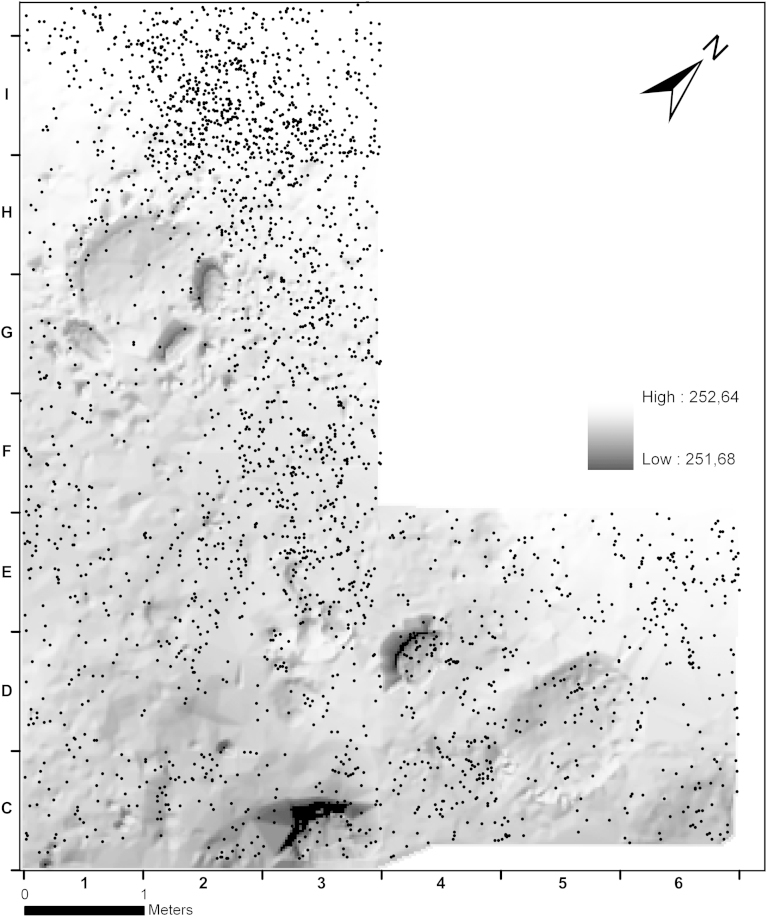
Krems-Wachtberg: Surface model of in situ base of layer AH 4.4 with artefact distribution of AH 4.11 (graph: Prehistoric Commission, Austrian Academy of Sciences).

Pits adjoining the hearth to the south and east are directly related to the occupation level AH 4.4 (Fig. 1). Pits 3 and 7 are assigned to hearth phase I, pit 6 is assigned to hearth phase II.

The interpretation of the Krems-Wachtberg site as a base camp is backed by the occurrence of two burials (burial 1 and burial 2) as well as the artefact inventory. The lithic, bone, ivory and “ceramic” industries including mobile art and objects of personal adornment suggest, together with the existing ^14^C-dates (Einwögerer et al., 2009; Table 1), a chrono-cultural attribution to Pavlovian sites (Svoboda, 2007, 204–207; Einwögerer et al., 2008, 19). Recent investigations regarding seasonality of the faunal material of AH 4.4 point to an occupation in spring (Fladerer et al., 2014).

**Table 1:** Siliceous raw material spectrum in layers AH 4.4 and 4.11.

Raw material (RM)	RM group	AH 4.4		AH 4.11	
Chert	1	1655	24.2%	858	19.6%
Radiolarite	2	2083	30.4%	1617	36.9%
Chalcedony	3	48	0.7%	30	0.7%
Quarzite	4	74	1.1%	117	2.7%
Siliceous Limestone	5	1971	28.8%	1221	27.8%
Spiculite	7	856	12.5%	410	9.3%
Flint	9	55	0.8%	58	1.3%
Vein Quarz	11	1	0.0%	2	0.0%
Rock Crystal	12	0	0.0%	1	0.0%
Jasper	13	4	0.1%	7	0.2%
Spongiolite	14	97	1.4%	67	1.5%

		6844	100.0%	4388	100.0%

The following discussion compares the characteristics, stratigraphy and spatial relationship of layers AH 4.4 to AH 4.11.

## 2 Chrono-cultural attribution based on artefact morphology

The analysis relies on a data base of 10,473 stone artefacts above 10 mm in size, of which 61% (*n* = 6414) belong to AH 4.4 and 39% (*n* = 4059) to AH 4.11. The excavations in 2005–2008 cover an area of more than 30 m^2^ (not including a salvage excavation in autumn 2008). The material recovered since 2009 is not included in this study.

### 2.1 Lithic raw material

The siliceous raw material derives mostly from local provenance. The majority was sourced from the nearby gravel of the rivers Danube and Krems, located approximately 1 km from Krems-Wachtberg. Radiolarite, siliceous limestone and chert are the most common materials. Furthermore, angular chalcedony and jasper occur, deriving from sources in the nearby Waldviertel (Northwestern Lower Austria). Imported flint as well as quarzite of lower quality is far less numerous (Table 1). The raw material is divided into groups (RM, e.g. chert = group 1) and further into raw material units (RMU, e.g. 1_7).

The distribution of lithic raw material in both stratigraphic layers AH 4.4 and AH 4.11 is similar. No distinct chaîne opératoire within the different raw material units (RMU) of each horizon were present.

The raw material debitage of the site is documented by debris with natural surfaces, reduction flakes with surface or rests of reduction platforms and cores (Brandl and Reiter, 2008, 47). The surface is considered as the outermost part of rock, modified by natural/environmental influence.

### 2.2 Cores

The occupational horizon AH 4.4 provided a total of 67 cores (1% of the stone artefacts), whereas 62 cores were derive from AH 4.11 (1.5%). Nodules were mostly tested and reduction faces prepared before they were brought into the camp. Due to the generally poor quality of the nodules, more than half of the cores are core debris, leaving 34 nuclei for horizon AH 4.4 and 35 nuclei for horizon 4.11.

Most cores are complete, and up to 90% were sourced from the nearby gravels. The domination of flaked cores (2/3 of the cores; Fig. 2, 17) is reflected by the analysis of the debitage material. A controlled blade reduction strategy was attempted, but it is discernible on some cores that blades terminated in hinges, due to the low raw material quality.

The reduction of nuclei was mostly performed in a unipolar manner (Fig. 2, 16), though they were turned more often in AH 4.11 than in AH 4.4 (Table 2). Optimization of the exploitation of the nodules was attempted, as much as the unipolar technique allows. Furthermore, the striking platforms of the nuclei in AH 4.4 were reduced more often compared to the ones in AH 4.11, which might consequently have decreased the necessity to turn the cores frequently (Table 3).

**Table 2:** Core reduction in layers AH 4.4 and 4.11.

Core reduction	AH 4.4	AH 4.11
Unidirectional	22	17
Bidirectional	4	4
Opposite	1	–
Rotated	7	14

	34	35

**Table 3:** Reduction of striking platform edges in layers AH 4.4 and 4.11.

Reduction of striking platform edge	AH 4.4	AH 4.11
None	17	27
1 striking platform	15	7
2 striking platforms	2	1

	34	35

It is not surprising that fine-grained material was used more economically. A bifacial reduction strategy is more frequently used within these RMU. Moreover, imported flint (RMU 9_1) was used for the production of burins. Debris of that RMU was further reduced. The fine-grained siliceous material was regarded as valuable and therefore intensely exploited (Ziehaus, 2007, 100).

### 2.3 Lithic debitage

The following presents some aspects of the lithic debitage production. The bulk of the debitage assemblage consists of flakes, debris and blades/bladelets (about one-third each). Furthermore, a low percentage of preparation debitage (3%, including crested blades and flakes), cores (more than 1%) and discarded burin spalls (less than 1%) was noted. The debitage assemblages of AH 4.4 and 4.11 both show higher proportions of flakes compared to blades, but the percentage of blades is higher by 5.2% for AH 4.11 (Fig. 3).

The preparation of edges by dorsal reduction was documented in both horizons more often on blades than on flakes. There is a difference in the reduction rate for each horizon. In AH 4.11, blades are more frequently reduced (64%) compared to AH 4.4 (56%). The same tendency can be confirmed regarding the flakes in both layers, though the difference between AH 4.11 (45%) and AH 4.4 (43%) is not as significant as with blades.

In contrast to the frequent dorsal reduction, preparation of the striking platform by faceting is observed more rarely. Interestingly, it is more common on flakes (AH 4.11: 23%; AH 4.4: 19%) than on blades (AH 4.11: 17%; AH 4.4: 14%). These corrections presumably indicate frequent turning of the cores and therefore confirm intensive exploitation. Plane striking platforms dominate with blades (AH 4.11: 76%; AH 4.4: 77%) as well as flakes (AH 4.11: 61%; AH 4.4: 59%).

The predominant direction of dorsal knapping scar patterns on blades and flakes confirm unipolar core reduction. In both stratigraphic layers, almost 90% of the blades and 80% of the flakes showed unipolar dorsal scar patterns. Bipolar scar patterns are far less common (<1%) on both types of debitage, though slightly more commonly observed on blades (AH 4.4: 2.9%; AH 4.11: 3.7%) than on flakes (AH 4.4: 1.6%; AH 4.11: 2%). Blades and flakes from cores with multiple percussion platforms show vertical and horizontal knapping scar patterns. In both layers, this pattern is more evident on flakes (11%) than on blades (AH 4.4: 5.1%; AH 4.11: 6.5%). The comparison of blades and flakes in this regard shows that flakes are products of a certain preparation technique. Consequently, there is a higher percentage of flakes with a primary surface and no scar patterns – 7% in contrast to blades with only 2.3% (AH 4.4) and 1.4% (AH 4.11). It is apparent that initial debitage stages have taken place on site.

A similar debitage production for both horizons may indicate contamination of material from AH 4.4 into AH 4.11. Minor differences regarding the proportions of blades and flakes with dorsal reduction could support the observation that AH 4.11 partly derives from another source (see above).

### 2.4 Lithic tool varieties

With only 121 tools among the 10,473 artefacts of the lithic inventory, the proportion is only 1.2% (Table 4). The wide range of lithic artefacts has abundant backed bladelets and laterally retouched artefacts which account for 1/4 of the assemblage. Backed points, microgravettes, different types of burins, endscrapers and truncated pieces make up 7–11% of the tools. Other types, including notched, splintered, denticulated pieces or discard and combination tools are only represented by single examples.

**Table 4:** Lithic tools in layers AH 4.4 and 4.11.

Tools	AH 4.4	AH 4.11
Endscraper	7	5
Truncation	6	3
Burin	6	7
Backed point	1	2
Microgravette	3	5
Point	–	1
Backed bladelet	16	15
Retouched piece	13	16
Pointed blade	–	1
Notched piece	1	1
Microdenticulate	2	2
Microlith	1	–
Splintered piece	1	–
Combination tool	1	1
Discard	1	3

	59	62

The high number of backed tools and the existence of microgravettes as well as microdenticulates (Fig. 2, 1–15) emphasize the attribution of this lithic assemblage to the Gravettian, respectively Pavlovian (Soffer, 1999, 59–76; Svoboda, 1996, 283–301; Svoboda et al., 1999, 197–218). More than half of these types of tools have wear usage, including edge damage or edge abrasion. Among them are several pieces with distinct splinters, which suggest discarding after usage or damage.

About 43% (52 pieces) can be considered as “microlithic” tools considering their small width (less than 75 mm; Bartošíková, 2005, 112–133; Škrdla, 2005, 47) which emphasises their attribution to the Pavlovian. Backed or denticulated tools (42 artefacts) dominate among those “microlithic” forms. Only 7 tools of this category have a width of more than 75 mm, especially the pointed forms.

Looking at the amount of siliceous tools in both layers, AH 4.4 with 0.9% has fewer tools than AH 4.11 with 1.5%. The low amount of tools, which seems to be unusual for a Gravettian base camp, could be due to different reasons. On the one hand, it could reflect the poor quality of the raw material, which requires a more careful handling of specially modified artefacts. Tools most likely were taken along as the camp was abandoned, and the ones left behind show intensive traces of usage, as in the case of endscrapers with rounded edges. On the other hand, it has to be considered that only part of the site has been excavated, or other areas of the site might possibly be eroded.

## 3 Intra-site chrono-stratigraphy

### 3.1 AH 4.4 and 4.11: living floor and translocated layer

First re-fittings provide evidence that both layers are from the same provenience (Ziehaus, 2007, 92–100.). The refitted artefacts were found about 4 m apart. The ^14^C-dates of AH 4.11, which are partly older than for AH 4.4 (e.g. VERA-3932: 28,300 ± 270 BP, Einwögerer et al., 2009, Table 1) indicate that the material may in part derive from an older camp site located further up the hill with a tool assemblage that is mostly coherent with AH 4.4.

An attempt was made to filter older reduction techniques by a technological analysis. A few technological differences can be pointed out, but in general the archaeological inventories of AH 4.4 and 4.11 do not differ significantly.

The following Aurignacian and Gravettian sites of eastern Austria were compared: Gravettian assemblages as Krems-Wachtberg 2005 (Ziehaus, 2007), Krems-Wachtberg 1930 (Einwögerer, 2000, 101–103), Krems-Hundssteig 2000–2002 (Einwögerer and Simon, 2008) and Langenlois A (Einwögerer, in press); and two Austrian Aurignacien sites, Willendorf II/3 (Nigst, 2010, 81–99) and Senftenberg (Hinterwallner, 2006). One significant difference between these two Palaeolithic periods in the investigated region is a better result regarding the production of blades due to an improved reduction strategy during the Gravettian. The high amount of blades in AH 4.4 clearly suggests attribution to the Gravettian. Nevertheless, the preferred unipolar core reduction technique did not change from the Aurignacian to the Gravettian and seems to be related to the low quality of the local cobbles. In this geographical area, research on reduction techniques can therefore only give vague answers concerning a possible older provenance of some artefacts in AH 4.11.

### 3.2 Phases of hearth use

The central hearth complex in AH 4.4 consists of hearth 1 and the three adjacent pits 3, 6 and 7. Because the hearth functioned as a sediment trap, it is one of the features where a chronological differentiation within the living floor is possible. The stratigraphy of the hearth enables a distinction of three main phases (see above).

The raw material throughout the hearth’s phases shows no difference to the material outside the complex. Furthermore, the raw material quality remains similar in all phases. The procured raw materials are mainly chert (RMU 1_xx) and radiolarite (RMU 2_xx) as well as high quality siliceous limestone (RMU 5_3).

There is also no indication for a variation of the degree of alteration by fire of the raw material units among the phases. The presumption that more intensely burnt artefacts, due to a longer exposure to heat, would be found in the lower strata of the hearth cannot be confirmed.

A total of 2458 pieces of siliceous artefacts were found within the hearth, of which half show alteration by fire impact (Fig. 4). Although 48% of the lithics belong to phase II, the proportion of artefacts with fire impact was only 35% compared to 59% in phase I and 54% in phase III. This may imply a varying usage pattern of the hearth in phase II.

According to expectations, the amount of artefacts modified by heat outside the hearth (AH 4.4 exclusive hearth 1 and pits) is very low, at 4%. In contrast the high amount of burnt artefacts (about 50%) in the pits underlines their direct connection to the hearth.

The comparison of the occupation layer AH 4.4 of hearth 1 with adjacent pits to the living floor AH 4.4 apart from the hearth complex show no significant difference in regards of the lithic debitage. The percentage of debris in particular is almost identical. Blades and flake blanks tend to have been discarded less often into the hearth than in the periphery. Furthermore, the distribution of lithic debitage in phases I-III reveals an increase of the lithic debitage coherent with a decrease of debris in the course of the occupation (Fig. 5).

The proportion of artefacts with modifications by heat in hearth 1 is higher than outside the hearth complex. This includes tools (Table 5): half of the 26 tools in the hearth complex are burnt. In layer AH 4.4 without the hearth, the proportion is only 7%.

**Table 5:** Tools per phase in hearth 1.

Tools	Phase I	pit 3	Phase II	Phase III
Endscraper		1	4	
Truncation		1		
Burin	1		2	
Microgravette				1
Backed bladelet	3	1	2	
Retouched piece	2		3	1
Notched piece			1	
Microlith		1		
Splintered piece		1		
Discard			1	

	6	5	13	2

In phase III, only 2 pieces, both without heat modification, were found. The other 24 tools (both burnt and unburnt) are equally distributed among phases I and II. In pit 3, which belongs to phase I, there is a noticeable amount of 5 tools (almost 5% of the 104 siliceous artefacts). No tools were found in pits 6 and 7 (pit 6: *n* = 62 and pit 7: *n* = 34 lithics).

A total, of 44% of all modified stone artefacts that were excavated in layer AH 4.4 have a spatial affiliation to the hearth. Furthermore, there is a dominantly high proportion (35% of the tools inside the hearth complex) of truncated types or composite tools such as backed bladelets and backed points, including their production waste.

## 4 Spatial distribution in AH 4.4

The surface model illustrates the in situ base of the stratigraphic layer AH 4.4 (for description of the features, see Simon et al., 2014). The lithic find assemblage is plotted onto the model, showing the highest visible density of artefacts in the area of hearth 1 (Fig. 6). Another large concentration (c1) lies east of the hearth. A smaller concentration is situated in the direct northwestern neighbourhood (c2) and another less evident one north of it (c3). Even though the spatial distribution shows only a fractional part of the site, a clear decrease of the assemblage distribution to the south and also to the west and northwest of the hearth can be identified. The square meters furthest to the north-east (I2 and I3) show a stratigraphic discontinuity because AH 4.4, which is already fading out in this area, had originally not been recognized as a separate horizon.

The lithics of archaeological horizon AH 4.11 show a wider distribution than those from AH 4.4 - especially in the north-east (Fig. 7). It appears as if the hearth’s sediments built up to a shallow mound formed an obstacle. This could be related either to taphonomic processes or constructional issues. The distribution of artefacts in AH 4.11 east of the hearth reflects concentration c1 of AH 4.4. This supports a mixing of AH 4.11 with the uppermost parts of AH 4.4 as an aspect of vertical translocation.

### 4.1 Spatial distribution of the raw material

**Fig. 8:**
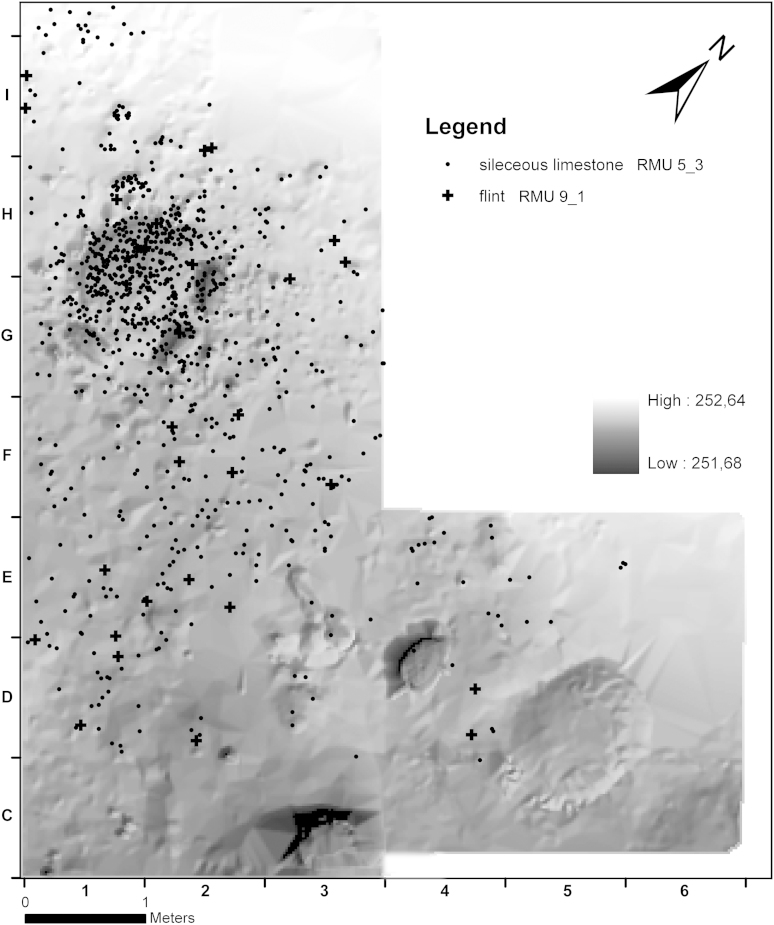
Krems-Wachtberg: Raw materials RMU 5_3 and 9_1 in layer AH 4.4 (graph: Prehistoric Commission, Austrian Academy of Sciences).

The investigation of the spatial distribution of artefacts was based on well classifiable raw material units in order to determine possible zones of activity around the hearth area within the in situ horizon AH 4.4. One of the main raw materials is siliceous limestone (RM 5, *n* = 1971). The largest raw material unit RMU 5_3 in this group (*n* = 1822) is representative for the distribution of all lithics in AH 4.4. Within the hearth, the degree of heat impact does not vary during the hearth phases I to III. However, in each phase the more heavily burnt stone artefacts are found mostly in the centre of the find concentration. This provides evidence for a higher or longer lasting heat impact in the centre. Another example of a raw material unit with a representative distribution, although much scarcer (*n* = 57), is the imported flint RMU 9_1 (Fig. 8).

In contrast, other RMUs are concentrated in specific areas of AH 4.4 and reflect selected stages of the chaîne opératoire. For instance, RMU 1_7 (chert, *n* = 131) represents a split concentration in the area east of hearth 1 (c1 and c3). Half of its debitage consists of blades, and one-third are flakes. Besides a burin spall only 3 pieces of preparation debitage, very few debris and no cores were found. Three-quarters of the artefacts are decortified.

The majority of artefacts of RMU 14_1 (spongiolite, *n* = 97) are blades or flakes which are basically scattered over the whole area, but show faint concentrations north and east of the hearth. The same applies for RMU 7_5 (spiculite, *n* = 47). Only limited debris and preparation debitage in favour of more blades and flakes were excavated. The concentration of the assemblage was retrieved south of square metre F1.

Debitage of RMU 1_32 (chert, *n* = 54) is mostly represented by debris, some blades and flakes. The material is entirely burnt. With very few exceptions it occurs only inside hearth 1, evenly distributed throughout all phases.

The data suggest that RMU 5_3 and 9_1 had been used in the earliest phase and are hence scattered over the whole area of the living floor. Dispersal of material increases with the duration of occupation (Vaquero and Pastó, 2001, 1215).

**Fig. 9:**
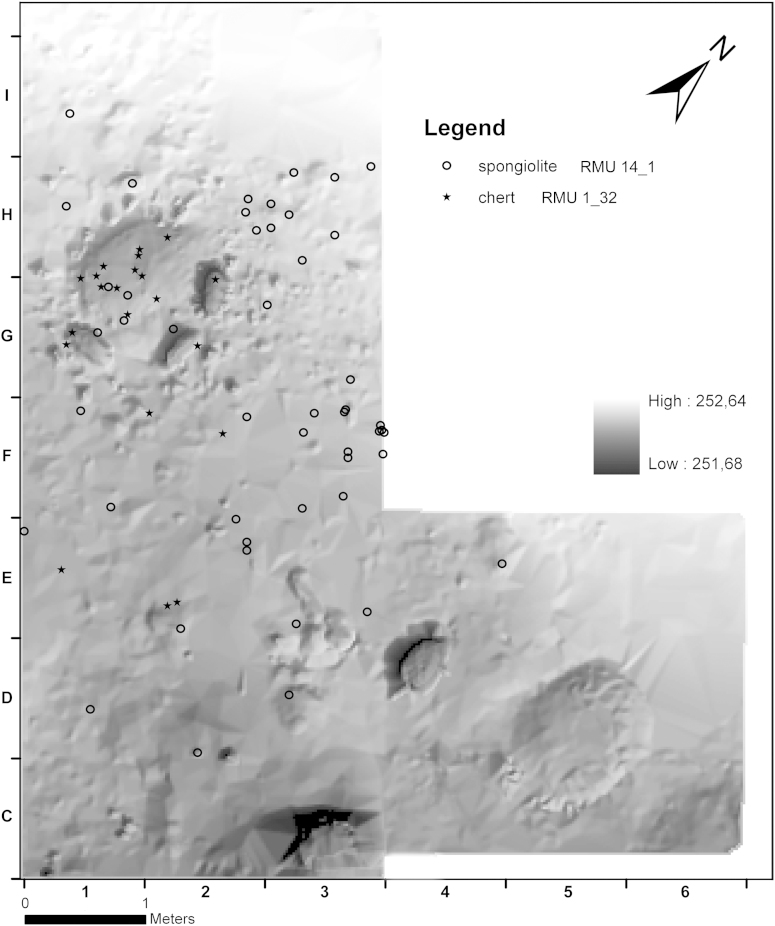
Krems-Wachtberg: Raw materials RMU 1_7 and 14_1 in layer AH 4.4 (graph: Prehistoric Commission, Austrian Academy of Sciences).

**Fig. 10:**
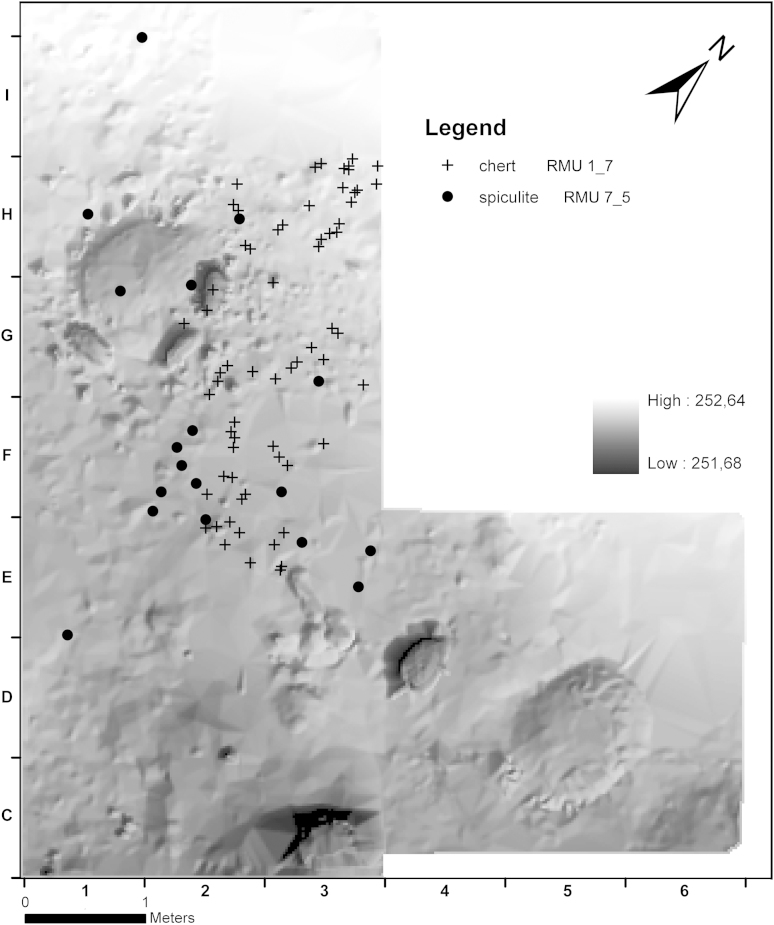
Krems-Wachtberg: Raw materials RMU 7_5 and 1_32 in layer AH 4.4 (graph: Prehistoric Commission, Austrian Academy of Sciences).

The preparation of chert RMU 1_7 must have taken place somewhere else and further reduction happened in the vicinity of the hearth. In contrast, nodules of spongiolite and spiculite (RMU 14_1 and 7_5) were reduced outside the excavated area and end products transported to the hearth. It is most likely that artefacts of chert RMU 1_32 were discarded in the hearth in each single phase. These concentrations provide evidence for single events, presumably more towards the end of the occupation (Fig. 9, Fig. 10).

### 4.2 Activity zones within living floor AH 4.4

The distribution of each type of blank reflects the general spatial distribution of all siliceous artefacts without any specific accumulation/concentration. Only cores are concentrated in the hearth area and in the south outside of concentration c1.

**Fig. 11:**
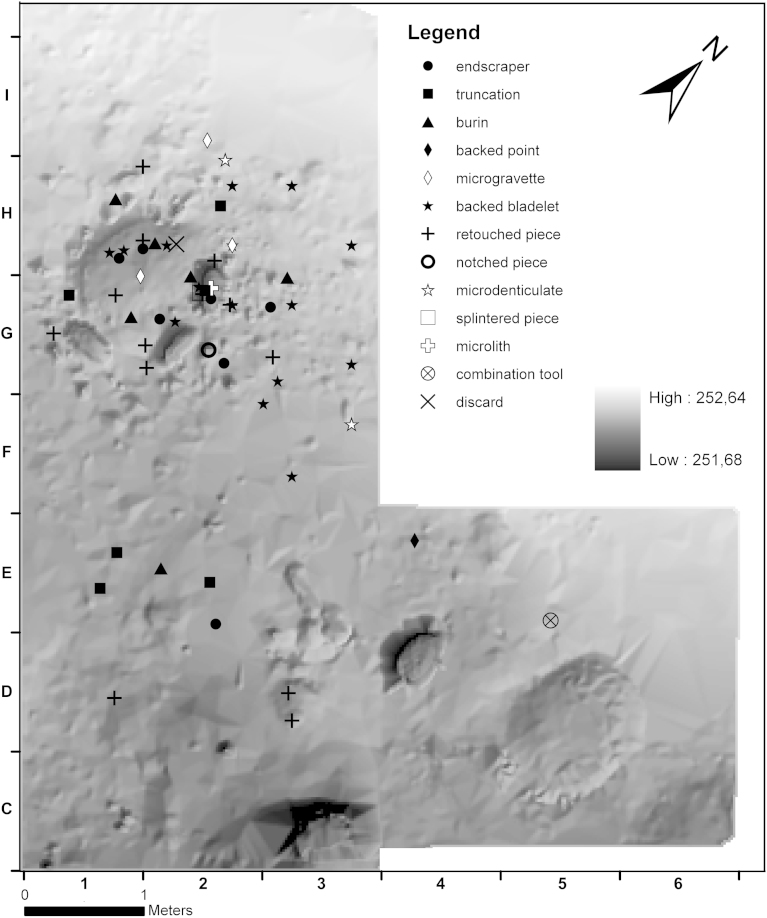
Krems-Wachtberg: Spatial distribution of tools in layer AH 4.4 (graph: Prehistoric Commission, Austrian Academy of Sciences).

As mentioned above, tools show a strong affinity to the hearth (Fig. 11). An accumulation of modified stone artefacts is located not only inside the hearth complex but also in its eastern periphery. South of c1, separated by an area almost without retouched artefacts, there is another loose concentration. The artefact concentration c1 itself bears only few tools in its periphery. Backed bladelets occur only in or around the hearth. The same applies to endscrapers, with one exception in the south. Truncations and retouched pieces are found in and around the hearth as well as in the southern “tool-concentration”. Activity zones related to the exclusive use of certain tool types cannot be specified.

### 4.3 Phases of the hearth feature

**Fig. 12:**
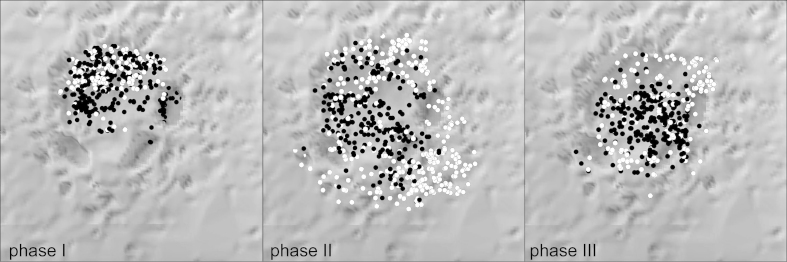
Krems-Wachtberg: Distribution of artefacts with (black) and without (white) fire impact in the phases 1–3 of hearth 1 (graph: Prehistoric Commission, Austrian Academy of Sciences).

A shallow depression had been dug to accommodate the hearth. In its initial phase the fireplace is limited to this feature and to the pits 3 and 7 (Fig. 12). The unburnt stone artefacts are concentrated rather in the northwestern half of the depression. During the second phase, the hearth increased in area significantly to the southeast and was superimposed on pits 3 and 7. The possibility that pits 3 and 7 were in use later than phase I can therefore be excluded, and pit 6 was utilised. The main concentrations of the unburnt stone artefacts are located further east and in the western periphery. In the final phase III, the distribution of the assemblage is restricted to the central section, above the southeast part of the depression and in the area above pit 7. This spatial limitation is possibly due to erosion, or indicates a final brief event.

## 5 Results and discussion

These studies confirm that Krems-Wachtberg had been used as a seasonal base camp based on the existence of all stages of lithic reduction sequences. The lithic debitage with a high proportion of blades of about 25% shows that the production of blades had been targeted. The production of backed bladelets, the occurrence of typical microdenticulates and microgravettes together with other aspects like the burials, evident features and spatial patterns, bone, ivory and “ceramic” industries, red ochre, objects of personal adornment and mobile art suggest a chrono-cultural attribution to the Pavlovian (Svoboda, 1996, 283–301; Svoboda et al., 1999, 197–218).

The field observations considering the relationship of layers AH 4.4 and 4.11 within the stratigraphic complex AH 4 can be confirmed. A detailed classification and genetic explanation of the translocated AH 4.11 must remain preliminary and should be investigated further. Similar raw material units and reduction techniques and a congruent spatial distribution pattern of both stratigraphic units indicate a contamination of find material from AH 4.4 into 4.11. Nevertheless, AH 4.11 shows a slightly varying lithic artefact morphology compared to AH 4.4. A technological attribution of layer AH 4.11 to an older chronological period (as implied by several ^14^C-dates) cannot be confirmed. AH 4.11 can be interpreted as a horizontally translocated horizon containing material from an occupational layer located further uphill which has taken along the upper part of AH 4.4 (Händel et al., 2014).

One focal point of this paper concentrates on modelling the phases of use for hearth 1. Each phase of the hearth shows a similar spectrum of raw material and reduction technology. Differences, however, are indicated by the proportions of blanks. For the initial phase I, an intensive lithic debitage production including discarding of waste material (high proportion of debris and tools) was observed. At the beginning of the settlement, already tested raw material nodules were brought into the camp and the raw material waste was discarded. The comparatively high proportion of 5% tools in pit 3 suggests a more intense tool production and/or discarding at the beginning of the use of the hearth.

Phase II represents the main utilization phase of the hearth, which also increased in size. This phase contains the major proportion of artefacts. Gradually, the percentage of waste material declined, in spite of the constant lesser quality of the procured raw material. In phase III, standardized blades and tools were rarely discarded in the hearth, but most likely were taken along when the camp was abandoned.

It can be assumed that the implements’ proximity to the hearth is due to activities connected to hafting and retooling (Keeley, 1987, 89–96). After the active initial phases I and II, there is little evidence for tool production in phase III. As tools had only rarely been discarded, the duration of phase III could have been relatively short. Perhaps this can be interpreted as the end of the occupational phase.

The change in firing material is possibly related to a functional shift. Had bones been burnt because the available firewood ran short? Was all the waste burned before the camp was abandoned to keep scavengers away from the burials? Or does the burning of bones indicate another fuel strategy where low temperature and longer lasting fires were needed?

A chronological depth is also supported by the spatial distribution of RMU. Although some raw material units had been used since the beginning of the occupation of the site and were therefore scattered all over the camp, other concentrations show individual events towards the end of the settlement.

The Krems-Wachtberg excavation involved a multi-phased and multi-functional hearth feature. The investigation of the lithic debitage assemblage, lithic tool production, faunal remains regarding the processing of food and creation of art objects made of formed and fired sediment, just to name a few, all demonstrate the significance of the hearth in the life of prehistoric hunter-gatherers.
